# Epileptic Phenotypes Associated With SNAREs and Related Synaptic Vesicle Exocytosis Machinery

**DOI:** 10.3389/fneur.2021.806506

**Published:** 2022-01-13

**Authors:** Elisa Cali, Clarissa Rocca, Vincenzo Salpietro, Henry Houlden

**Affiliations:** MRC Centre for Neuromuscular Diseases, UCL Queen Square Institute of Neurology, London, United Kingdom

**Keywords:** SNARE (soluble N-ethylmaleimide-sensitive fusion protein attachment protein receptor), epilepsy, seizures, mutations, vesicle fusion, epileptic encephalopathies

## Abstract

SNAREs (soluble N-ethylmaleimide sensitive factor attachment protein receptor) are an heterogeneous family of proteins that, together with their key regulators, are implicated in synaptic vesicle exocytosis and synaptic transmission. SNAREs represent the core component of this protein complex. Although the specific mechanisms of the SNARE machinery is still not completely uncovered, studies in recent years have provided a clearer understanding of the interactions regulating the essential fusion machinery for neurotransmitter release. Mutations in genes encoding SNARE proteins or SNARE complex associated proteins have been associated with a variable spectrum of neurological conditions that have been recently defined as “SNAREopathies.” These include neurodevelopmental disorder, autism spectrum disorder (ASD), movement disorders, seizures and epileptiform abnormalities. The SNARE phenotypic spectrum associated with seizures ranges from simple febrile seizures and infantile spasms, to severe early-onset epileptic encephalopathies. Our study aims to review and delineate the epileptic phenotypes associated with dysregulation of synaptic vesicle exocytosis and transmission, focusing on the main proteins of the SNARE core complex (STX1B, VAMP2, SNAP25), tethering complex (STXBP1), and related downstream regulators.

## Introduction

Epilepsy is defined as a large heterogeneous group of diseases in which individuals have an enduring predisposition to seizures (1), characterized by many different seizure types and epilepsy syndromes (2, 3). The term “epileptic encephalopathy” refers to a group of disorders in which unremitting epileptic activity contributes to progressive cerebral dysfunction (4). The epilepsies have a wide range of etiologies, which include genetic, metabolic, immune, and inflammatory factors; acquired or congenital brain abnormalities, infections, trauma or hypoxic-ischemic insults due to brain injuries (3). A genetic cause has been found in more than 50% of epilepsy phenotypes, particularly in developmental epileptic encephalopathies (5, 6). Pathogenic variants in genes encoding several voltage-gated K+, Na+, and Ca^2+^ channels subunits are the most common genetic cause of epileptic encephalopathies, accounting for a group of diseases defined as “channelopathies” (7). Another class of functionally related proteins have been discovered by James E. Rothman in 1994 and given the name SNARE (soluble N-ethylmaleimide sensitive factor attachment protein receptor) (8). Since then, several mutations in each subunit of the SNARE complex have been associated with an heterogenous group of neurological disorders, together referred to as SNAREopathies. SNAREs are a protein family involved in transport and release mechanisms of synaptic vesicles inside the neuron. They mediate the fusion of membranes by localizing both at the vesicular and target membrane. The core SNARE machinery consists of VAMP2 (synaptobrevin-2), the only vesicular binding SNARE (v-SNARE) of the complex and a combination of target membrane SNARE proteins (t-SNAREs): syntaxin1-A (STX1A) and synaptosomal-associated protein 25 kD (SNAP25) (9). The assembly of the SNARE machinery is carefully arranged by the assembly complex, which is composed of MUNC18-1 and MUNC13-1 and plays an essential role in the fusion of synaptic vesicles (10). Pathogenic biallelic and monoallelic variants disrupting proteins of the SNARE complex and associated regulators are a known cause for neurodevelopmental disorders consisting of an overlapping phenotype of developmental delay (DD), intellectual disability (ID), movement disorders, and epilepsy. The SNARE phenotypic spectrum associated with seizures ranges from simple febrile seizures and infantile spasms, to severe early-onset epileptic encephalopathies. Here we review the epileptic phenotypes associated with dysregulation of synaptic vesicle exocytosis and transmission, focusing on the main proteins of the SNARE core complex (STX1B, VAMP2, SNAP25), assembly complex (STXBP1, UNC13A), and related downstream regulators.

## Proteins of the Main Snare Complex

### STX1B

Sintaxin-1B (*STX1B*) codes for a presynaptic plasma membrane protein that belongs to the syntaxins family and is predominantly expressed in neurons. *STX1B* is part of the SNARE complex and its main role is to mediate the calcium-dependent synaptic vesicle release (11, 12). The crucial role of *STX1B* is underlined by studies performed on animal models. In fact, *STX1B* KO mice presented with impaired brain development, disrupted motor coordination, and only survived the first 14 days. Hippocampal cell cultures viability was lower compared to controls (13). Interestingly, heterozygous *STX1B* presented with a less severe phenotype (14), while heterozygous zebrafish models showed jerks and paroxysmal movements. Epileptic episodes were observed in approximately 50% of the animals, with increased events associated with higher temperature which is in concordance with the occurrence of febrile seizures (15).

A total of 40 different heterozygous or *denovo* mutations in *STX1B* have been described so far. These included 4 missense, 2 indels, 6 nonsense, 7 frameshift, 7 splice variants and 4 large indels (Table 1). Most of these mutations were predicted to cause haploinsufficiency of *STX1B*, resulting in early termination of the protein. Sixty-two individuals have been described so far and the mainly reported phenotype was epilepsy (Table 2) (15–28). Wolking et al. (23) divides STX1B-associated epileptic phenotypes in four different groups: (1) benign epilepsy syndrome with febrile and afebrile seizures corresponding to “genetic epilepsies with febrile seizures plus,” (2) “genetic generalized epilepsy” phenotype, (3) “developmental and epileptic encephalopathy” syndrome with refractory seizures and moderate to severe developmental deficits, and (4) focal epilepsy phenotype. Seizures have been described in almost all the individuals (61/62, 98%) and, where specified, they were the main symptom at onset in the majority of the individuals (43/48, 90%). Generalized seizures were the most common type of onset, presenting in 53 individuals (53/57, 93%), while focal-onset seizures occurred in 14 individuals (14/39, 36%). Seizure type ranged from tonic-clonic seizures (40/56, 71%) absence seizures (16/47, 34%), tonic or atonic seizures (24/56, 43%) to myoclonic seizures (16/30, 53%). Infantile spasms and status epilepticus were infrequent, both being reported in only two individuals. Out of the 47 electroencephalography recordings available, 42 showed epileptiform or non-epileptiform abnormalities (42/47, 89%).

**Table 1:** Mutations in SNARE proteins encoding genes.

**Type of mutation**									
**Gene**	**Inheritance**	**Zygosity**	**Missense**	**Nonsense**	**Frameshift**	**Splicing**	**Indel**	**Large indels**	**Associated OMIM number**
VAMP2	AD	*Denovo*	7/11	1/11	1/11	–	2/11	–	185881
STX1B	AD	Heterozygous, Denovo	14/40	6/40	7/40	7/40	2/40	4/40	601485
SNAP25	AD	Denovo	15/19	2/19	–	2/19	–	–	600322
STXBP1	AD, AR	Heterozygous, Homozygous, Denovo	60/209	30/209	42/209	34/209	2/209	41/209	602926
MUNC13-1	AD, AR	homozygous, denovo	1/2	1/2	–	–	–	–	609894
GOSR2	AR	Homozygous, Compound Heterozygous	4/7	–	–	2/7	1/7	–	604027
SNAP29	AR	Homozygous, Compound Heterozygous	3/10	2/10	5/10	–	–	–	604027
STXBP5L	AR	Homozygous	1	–	–	–	–	–	609381
CPLX1	AR	Homozygous	1/3	2/3	–	–	–	–	605032

**Table 2:** Main phenotypes associated with mutations in the SNARE complex.

**Gene**	**Disorder (#OMIM)**	**MOI**	**Cases**	**Age of onset**	**Seizures**	**EEG discharges**	**Global developmental delay**	**Intellectual disability**	**Motor impairment**	**Movement disorders**	**Autism/behavioral abnormalities**	**MRI abnormalities**
**Main proteins**												
STX1B	Generalized epilepsy with febrile seizures plus, type 9 (#616172)	AD	62	Early infancy	61/62 (98%)	42/47(89%)	15/47 (32%)	23/59 (39%)	15/48 (31%)	2/51 (4%)	5/45 (11%)	5/27 (19%)
VAMP2	Neurodevelopmental disorder with hypotonia and autistic features with or without hyperkinetic movements (#618760)	AD	11	Early childhood	6/11 (~55%)	7/9 (~78%)	11/11 (100%)	9/9 (100%)	8/10 (80%)	06/11 (~55%)	9/9 (100%)	4/10 (40%)
STXBP1	Developmental and epileptic encephalopathy 4 (#612164)	AD	>400	Early infancy	401/446 (~90%)	160/226 (~71%)	297/313 (~95%)	261/279 (~94%)	143/261 (55%)	130/261 (50%)	96/274 (35%)	111/257 (43%)
SNAP25	Myasthenic syndrome, congenital, 18; Developmental and epileptic encephalopathy (#616330)	AD	23	Early infancy to childhood onset	17/23 (73.9%)	15/16 (93.75%)	23/23 (100%)	23/23 (100%)	12/15 (80%)	5/21 (23.81%)	3/18 (16.67%)	6/21 (29%)
MUNC13-1	Not on OMIM	AR/AD	2	Early infancy	1/2 (50%)	2/2 (100%)	2/2 (100%)	2/2 (100%)	2/2 (100%)	1/2 (50%)	1/2 (50%)	1/2 (50%)
**Other associated proteins**												
CPLX1	Developmental and epileptic encephalopathy 63 (617976)	AR	5	Early infancy	5/5 (100%)	3/3 (100%)	5/5 (100%)	5/5 (100%)	3/3 (100%)	–	–	3/5 (60%)
STXBP5L	Not on OMIM	AR	2	Early infancy	2/2 (100%)	–	2/2 (100%)	–	–	1/2 (50%)	–	2/2 (100%)
SNAP29	Cerebral dysgenesis, neuropathy, ichthyosis, and palmoplantar keratoderma syndrome (609528)	AR	25	Late infancy/Childhood	9/25 (36%)	–	25/25 (100%)	25/25 (100%)	–	0/25 (0%)	–	21/22 (95%)
GOSR2	Epilepsy, progressive myoclonic 6 (#614018)	AR	34	Childhood	31/33(94%)	22/22 (100%)	6/26 (23%)	5/24 (20%)	32/32 (100%)	24/26 (93%)	–	5/16 (31%)
		**Seizures**	**EEG discharges**	**Seizures as first symptom at onset**	**Infantile spasms**	**Generalized onset**	**Tonic-clonic**	**Absence**	**Tonic and atonic**	**Myoclonic**	**Focal onset**	**Status epilepticus**
STX1B		61/62 (98%)	42/47(89%)	43/48 (90%)	2/54 (4%)	53/57 (93%)	40/56 (71%)	16/47 (34%)	24/56 (43%)	16/30 (53%)	14/39 (36%)	2/8 (25%)
VAMP2		6/11 (~55%)	7/9 (~78%)	0	3/6 (50%)	5/6 (83%)	2/5 (40%)	0	1/5 (20%)	1/5 (20%)	3/6 (50%)	2/6 (33%)
SNAP25		73.91%	93.75%	7/17 (41.18%)	5/17 (29.41%)	11/17(64.71%)	7/17 (41.18%)	6/17 (35.29%)	4/17 (23.53%)	4/17 (23.53%)	6/17 (35.29%)	1/17 (5.88%)
STXBP1		401/446 (~90%)	160/226 (~71%)	69/84 (82%)	162/260 (62%)	108/159 (68%)	77/258 (30%)	22/247 (9%)	84/246 (34%)	49/236 (21%)	140/274 (51%)	9/163 (6%)

Global developmental delay was documented in 15 individuals (15/47, 32%), and 23 individuals manifested various degrees of intellectual disability (23/59, 39%). Motor impairment, mainly ataxia, has been reported in 15 individuals (15/48, 31%). Behavioral or movement abnormalities were infrequent, accounting for 10% and 4% of the cases, respectively (5/45 and 2/51, respectively). When available, magnetic resonance imaging (MRI) was unremarkable for the majority of the cases (22/27, 81%).

### VAMP2

*VAMP2* encodes synaptobrevin-2, a major neuronal v-SNARE protein responsible for fusing synaptic vesicles at mammalian central nerve terminals (29, 30). *VAMP2* KO mice presented with abnormal body shape and died shortly after birth, brain abnormalities were not detected (31). Moreover, synaptic vesicle observed under the electron microscope from VAMP2 KO mice presented abnormal morphology and size (32).

*De novo* mutations in VAMP2 have hitherto been reported in 11 individuals with neurodevelopmental disorder (Table 1) (33–35). VAMP2 has been first described as a causative gene by Salpietro et al. (33), who reported 5 unrelated individuals with *de novo* heterozygous mutations. Simmons (34) and Sunaga (35) further expanded the cohort with 6 additional unrelated individuals carrying *de novo* heterozygous mutations in VAMP2. The mutations described so far include 7 missense, 2 indels, 1 nonsense and 1 frameshift. The entire cohort of variants is localized in the highly conserved SNARE motif.

All the affected individuals showed moderate to severe global developmental delay and intellectual disability (11/11; 100%). Behavioral abnormalities, including Autism Spectrum Disorder (ASD) or Rett-like features, were virtually present in all the reported cases (9/9; 100%). Movement abnormalities were present in 6 individuals (6/11, 55%) and included chorea (*n* = 3), dystonia (*n* = 1), myoclonic jerks (*n* = 1), tremor (*n* = 1) and hyperkinetic movements (*n* = 1). Even though seizures have only been reported in 6 individuals (6/11, 55%), electroencephalography (EEG) recordings, where available, showed abnormalities in 7 individuals (7/9; 78%). Generalized-onset seizures were the most prevalent type, occuring in 5 individuals (5/6, 83%). Among those, seizure type varied from tonic-clonic (*n* = 2), tonic/atonic (*n* = 1) and myoclonic (*n* = 1). Infantile spasms have been reported in 3 individuals (3/6, 50%), two of them in the West syndrome spectrum. Focal seizures were less frequent, occurring in 3 individuals (3/6; 50%). Two individuals experienced status epilepticus (2/6, 33%). Magnetic resonance imaging was unremarkable for 6 individuals, otherwise MRI findings included corpus callosum thinning or hypoplasia (*n* = 2), periventricular FLAIR hyperintensities (*n* = 1) or mild brain atrophy (*n* = 1) (Table 2). Of the 11 individuals with *de novo* variants, seven different missense variants were identified, in addition to two single residue deletions and two stop gains. All variants were absent from GnomAD database blablabla to date, it seems that no clear correlation between the type of variant and the phenotype has been identified.

### SNAP25

*SNAP25* encodes the SNAP-25 protein, a t-snare widely expressed in the brain which is localized both at the presynaptic nerve terminal as well as to neuronal membranes (36). SNAP-25 is distinctive in the SNARE complex as it lacks a transmembrane domain and contains two SNARE motifs separated by a linker region (37). When trying to generate KO *SNAP25* mice, it was observed that heterozygous mice did not present any significant differences in comparison to their wilt-type littermates. However, no homozygous *SNAP25-/-* were generated from the heterozygous crosses. Analysis of the homozygous fetuses revealed smaller size, absence of movements and blotchy appearance likely caused by vascular abnormalities of the skin. Morphology of the brain appeared normal (38). There have been 19 different *de novo* mutations identified so far in *SNAP25* across 23 patients (39). Out of the total number of variants, 14 were missense, 2 nonsense and 2 splice-site (Table 1). The mutations are localized in the SNARE motifs and are predicted to disrupt the SNARE complex, but thorough functional studies are yet to be performed.

All individuals presented with global developmental delay and intellectual disability (23/23, 100%), ranging between profound (4/20; 20%), severe (5/20; 25%), moderate (6/20; 30%), and mild (5/20; 25%). Regression was reported in five individuals (5/17; 29%) with three of them showing signs of regression with the onset of seizures. All individuals showed variable degree of motor delay, with motor impairment being more evident in 12 individuals (12/15, 80%). Seizures have been reported in 17 individuals (17/23, 74%). The age of seizure onset ranged between the 7th day of life to 12 years. In 14 individuals, the onset was before 2 years of age. Most individuals showed a broad spectrum of epileptic spasms, generalized and focal seizures. Generalized-onset or focal to generalized-onset seizures were the most frequently occuring, being reported in 11 individuals (11/17, 65%), while focal seizures were described in 6 individuals (6/17, 35%). Seizure spectrum included tonic-clonic (*n* = 7), absence (*n* = 6), tonic or atonic (*n* = 4), myoclonic (*n* = 4) seizures and epileptic spasms (*n* = 5). Status epilepticus was reported in only one individual. EEG abnormal findings, generally multifocal epileptic discharges and generalized spike wave discharges, were documented in 15 out of the 16 available records (15/16, 94%). Less frequently reported features were abnormal movements (5/21, 24%) and behavioral abnormalities (3/18, 17%). MRI was performed on 21 individuals and was unremarkable in 15 individuals (15/21, 71%) (Table 2).

## Snare Complex Assembly Factors

### MUNC18-1 (STXBP1)

MUNC18-1, also known as Syntaxin-binding protein-1 (*STXBP1*), belongs to the Sec1/Munc18 (SM) family. MUNC18-1, together with MUNC13-1, plays a crucial role in the SNARE complex assembly. More specifically, MUNC18-1 forms an inactive complex with syntaxin-1 to secure correct positioning of the latter. The formation of this complex is likely to represent the initial event for synaptic vesicle fusion (40).

KO mice have shown severe phenotype, dying immediately after birth and with no neurotransmission activity recorded. Moreover, degeneration was observed from cultured neurons from these mice (41, 42). Mutations in STXBP1 are the most commonly reported in literature, with a large difference in numbers compared to mutations in other SNARE proteins. So far, 163 distinct mutations and 41 large indels have been identified in *STXBP1*. The majority of this group is composed of 59 missenses, followed by 40 frameshift, 34 splice-site, 28 nonsense and 2 indels (Table 1). When missense variants are compared to all other types of mutations, there is no correlation to presence or absence of epilepsy. Cases reported to date were monoallelic, with the exception of two recently described siblings carrying a biallelic *STXBP1* L446F mutation (43).

More than 400 cases have been reported so far (19, 44–103). However, for many individuals, detailed clinical information was not available. Nevertheless, the key clinical findings in STXBP1-spectrum comprised global developmental delay and/or intellectual disability, seizures and variable presence of movement disorder, motor impairment or behavioral abnormalities. The most commonly described clinical feature is global developmental delay, presenting in 95% of the patients for whom information was available (297/313, 95%). The range of intellectual disability may vary: Stamberger et al. (56) reported that more than 80% of the individuals described until then presented with severe to profound intellectual disability.

Seizures were reported in 401 individuals (401/446, ~90%). A wide spectrum of seizure types was described in most individuals. Where specified, epileptic spasms frequently occurred at some stage during the disease course (162/260, ~63%). Other frequent seizure types were generalized-onset seizures (108/159, ~68%) and focal seizures (140/274, ~51%). Almost 90% of seizures occur in early infancy as the first symptom. When performed, EEG was reported abnormal in 71% of the cases (160/226, ~71%).

Other less commonly reported features include motor impairment [143/261 (55%)], movement disorders [130/261 (50%)] and behavioral abnormalities [96/274 (35%)]. In 146 of the 257 individuals for whom brain magnetic resonance imaging (MRI) was available, no abnormalities were documented (146/257, ~57%) (Table 2).

### MUNC13-1

MUNC13-1 is a protein encoded by *UNC13A* and is highly expressed in the hippocampus, cerebellum, cortex, striatum, and olfactory bulb (104). By binding both to synaptobrevin-2 and syntaxin-1, it aids in the formation of the SNARE complex, thus making synaptobrevin-2 more accessible by the MUNC18-1/syntaxin-1 formation (105).

Studies on MUNC13-1 KO mice have shown absence of evoked and spontaneous excitatory and inhibitory neurotransmitter release and synapses reduction in docked vesicles (106, 107). Until now, only two cases have been reported with mutations in MUNC13-1 (108, 109). A homozygous nonsense mutation (p.Gln102Ter) in UNC13A was identified in a girl with microcephaly, cortical hyperexcitability, fatal myasthenia, global developmental delay and intellectual disability. Seizures were not reported, but EEG showed abnormalities. MRI documented thinning of corpus callosum. Subsequently, a *de novo* heterozygous missense mutation was identified in a boy with dyskinetic movement disorder, developmental delay, intellectual disability, autism and ADHD, who also experienced febrile seizures.

### Other Associated Proteins

Epileptic phenotypes have also been associated with mutations in other SNARE-associated proteins, here we review GOSR2, SNAP29, STXBP5L, and CPLX1. The Golgi snap receptor complex member 2 (*GOSR2*), is part of a complex responsible for docking and fusion of newly synthesized proteins from the endoplasmic reticulum (110). To date, seven different biallelic variants have been reported in *GOSR2* (Table 1). All individuals share a similar phenotype with myoclonus epilepsy, ataxia, and usually relatively preserved cognition. SNAP29, acting in the autophagosome-lysosome fusion (111), has been found to harbor 10 distinct biallelic mutations (Table 1). Namely, 5 frameshifts, 2 nonsense and 3 missense (112). Mutations in SNAP29 have been linked to CEDNIK syndrome, whose clinical features include microcephaly, severe neurologic impairment, psychomotor retardation, failure to thrive, and facial dysmorphism, as well as palmoplantar keratoderma and late-onset ichthyosis (113). Global developmental delay and intellectual disability have been reported in all the affected individuals. Seizures are not very common and have been described in 9 of the 25 reported individuals (9/25, ~36%).

The function of *STXBP5L* is still not clear, but it has been observed it plays a role in the inhibition of the formation of the SNARE complex. Only one homozygous missense variant has been reported so far in two siblings with seizures, global developmental delay and MRI abnormalities (114, 115). Complexin-1 (*CPLX1*) is a neuronal protein of the SNARE complex, which contributes to vesicle fusing. To date, 2 nonsense and 1 missense biallelic mutations have been reported in the gene. Only five cases have been described so far, all presenting with seizures and global developmental delay (116, 117).

## Discussion

In the previous paragraphs, the epileptic syndromes associated with mutations or variants in the SNARE complex were briefly reviewed. Since the identification of SNARE proteins, many studies have focused on the role of the single molecules within the whole complex. Mutations in each subunit of the complex and in the related upstream and downstream regulators have been identified in a heterogeneous group of disorders, mostly neurological disorders. We focused on the association between the SNARE complex and seizures. Only four proteins involved in the synaptic vesicle fusion have not been linked to epileptic phenotypes: α-synuclein, synaptobrevin-1, and synaptotagmin-1 and -2.

Deficits in the subunits of the core complex (synaptobrevin-2, syntaxin-1B and SNAP-25), Munc18-1 and complexin-1 are mainly associated with an overlapping spectrum of developmental delay, intellectual disability, epilepsy, and movement disorders. Overall, the most reported feature is epilepsy, presenting in almost 90% of the individuals with a SNARE dysfunction. Particularly, mutations in syntaxin-1B are most associated with epileptic phenotypes. The type of seizures may vary from generalized tonic-clonic, myoclonic or absence seizures to focal seizures. Infantile spasms or West syndrome were also reported in association with SNARE dysfunction. The epileptic phenotypes associated with the main vesicle fusion machinery have been characterized in Table 2. Given the significant overlap in seizure semiology, it is not possible to differentiate the genetic cause based on seizure type. Global developmental delay and intellectual disability are also frequent features, presenting in 85% of the individuals. However, dysfunctions in STX1B and GOSR2 are less commonly associated with developmental delay, as most of the affected individuals don't show intellectual impairment. Motor impairment and movement abnormalities, including ataxia, gait abnormalities, tremor, hyperkinetic movements, chorea and myoclonus, are variably present, affecting almost half of the individuals. Behavioral abnormalities, comprising Autism Spectrum Disorder, Rett-like phenotypes and stereotypies, were less commonly documented in the cohort. Brain MRI was performed and reported as normal in more than 50% of the overall cohort. We comprehensively reported the total cohort of mutations identified so far in the genes that form the core SNARE complex and some associated proteins. The zygosity of genes in the core SNARE complex was heterozygous or *de novo*, indicating the crucial role of these proteins. Studies on animal models have confirmed this, by showing that homozygous KO animal models are either incompatible with life or severely affected, not surviving their first days. Therefore, it is likely that the early onset of the epileptic phenotype might be the consequence of the disruption of the neurotransmitters release machinery. Except for *STXBP1*, where only one biallelic variant was reported out of the total 209, all other discussed genes presented biallelic and monoallelic variants. In conclusion, we comprehensively described a cohort of more than 600 individuals affected with dysfunction in proteins of the SNARE complex or synaptic vesicle machinery. We illustrated the phenotypic spectrum of the SNARE-associated disorders and focused on the epileptic phenotypes. This review underlines the key role of SNARE proteins in the pathogenicity of epilepsy and the prevalence of this phenotype. Limitations of this study are mainly attributable to its retrospective nature. One important limitation has been the lack of precise information on the main phenotype and on the neuroradiological features, particularly when the mutation was reported in big cohort studies. Clinical features were summarized through percentages, but we cannot exclude the risk of under or overestimation. We reviewed the function of the main SNARE proteins, taking in consideration the consequence of their disruption in animal models. However, to better understand the pathways involved in these disease mechanisms, further functional studies will be required.

## Author Contributions

EC, CR, VS, and HH: conceptualization, writing—review, and editing. HH: funding acquisition. EC and CR: writing—original draft. All authors contributed to the article and approved the submitted version.

## Conflict of Interest

The authors declare that the research was conducted in the absence of any commercial or financial relationships that could be construed as a potential conflict of interest.

## Publisher's Note

All claims expressed in this article are solely those of the authors and do not necessarily represent those of their affiliated organizations, or those of the publisher, the editors and the reviewers. Any product that may be evaluated in this article, or claim that may be made by its manufacturer, is not guaranteed or endorsed by the publisher.
